# Secretion of soluble complement inhibitors factor H and factor H-like protein (FHL-1) by ovarian tumour cells

**DOI:** 10.1038/sj.bjc.6600614

**Published:** 2002-11-04

**Authors:** S Junnikkala, J Hakulinen, H Jarva, T Manuelian, L Bjørge, R Bützow, P F Zipfel, S Meri

**Affiliations:** Department of Bacteriology and Immunology, Haartman Institute, University Central Hospital, FIN-0014 Helsinki, Finland; Helsinki University Central Hospital, FIN-00014 Helsinki, Finland; Department of Microbiology and Immunology, The Gade Institute, Haukeland Hospital, N-5021 Bergen, Norway; Department of Obstetrics and Gynaecology, Haukeland Hospital, N-5021 Bergen, Norway; Department of Infectious Biology, Hans-Knoell-Institute, 07745 Jena, Germany; Department of Obstetrics and Gynaecology, Helsinki University Central Hospital, FIN-00014 Helsinki, Finland

**Keywords:** complement, ovarian tumour, factor H, FHL-1, natural immunity, monoclonal antibody

## Abstract

We observed that the soluble complement regulators factor H and factor H-like protein were abundantly present in ascites samples as well as in primary tumours of patients with ovarian cancer. RT–PCR and immunoblotting analyses showed that the two complement inhibitors were constitutively produced by the ovarian tumour cell lines SK-OV-3 and Caov-3, but not PA-1 or SW626 cells. The amounts of factor H-like protein secreted were equal to those of factor H. This is exceptional, because e.g. in normal human serum the concentration of factor H-like protein is below 1/10th of that of factor H. In ascites samples the mean level of factor H-like protein (130±55 μg ml^−1^) was 5.5-fold higher than in normal human serum (24±3 μg ml^−1^). Ovarian tumour cells thus preferentially synthesise factor H-like protein, the alternatively spliced short variant of factor H. The tumour cells were found to bind both ^125^I-labelled factor H and recombinant factor H-like protein to their surfaces. Surprisingly, the culture supernatants of all of the ovarian tumour cell lines studied, including those of PA-1 and SW626 that did not produce factor H/factor H-like protein, promoted factor I-mediated cleavage of C3b to inactive iC3b. Subsequently, the PA-1 and SW626 cell lines were found to secrete a soluble form of the membrane cofactor protein (CD46). Thus, our studies reveal two novel complement resistance mechanisms of ovarian tumour cells: (i) production of factor H-like protein and factor H and (ii) secretion of soluble membrane cofactor protein. Secretion of soluble complement inhibitors could protect ovarian tumour cells against humoral immune attack and pose an obstacle for therapy with monoclonal antibodies.

*British Journal of Cancer* (2002) **87**, 1119–1127. doi:10.1038/sj.bjc.6600614 www.bjcancer.com

© 2002 Cancer Research UK

## 

Ovarian cancer is the 6th most common malignant neoplasm among women worldwide, accounting for 4.3% of all female malignancies (Parkin *et al*, 1993). Despite advances in surgical and chemotherapeutic regimens and supportive care the overall 5-year survival is less than 40% (Bjørge *et al*, 1998). Most ovarian neoplasms are derived from the surface epithelium of the ovary and characteristically, the malignant cells remain confined to the abdominal cavity.

Since ovarian tumours grow within the peritoneal cavity for prolonged periods local adjuvant therapies could prove useful in this disease. Potential approaches include immunotherapy with monoclonal antibodies (mAbs), which could be directly injected into the peritoneal cavity. The mAbs could bind to individual tumour cells or their small clusters that remain after removal of the visible tumour masses. The antibodies, however, should be sufficiently specific for the tumour cells and recruit appropriate effector mechanisms including (i) the complement (C) system, (ii) the antibody-dependent cellular cytotoxicity (ADCC) and (iii) apoptosis.

Both normal and malignant human cells are protected against complement-mediated killing by specific membrane inhibitors. These include complement receptor 1 (CR1; CD35), membrane cofactor protein (MCP; CD46), decay accelerating factor (DAF; CD55) and protectin (CD59) (Morgan and Meri, 1994; Gorter and Meri, 1999). CR1 and MCP promote factor I (C3b inactivator) -mediated degradation of C3b (Fearon, 1979; Seya *et al*, 1986), whereas DAF and CR1 promote the decay of both the classical and alternative C pathway C3/C5 convertases (Fearon, 1979; Nicholson-Weller *et al*, 1982). CD59 acts by preventing formation of the membrane attack complex (MAC) on cell membranes (Sugita *et al*, 1988; Davies *et al*, 1989; Meri *et al*, 1990). CD59 has a key role in protecting tumour cells against C-mediated destruction (Hakulinen and Meri, 1994; Junnikkala *et al*, 1994; Bjørge *et al*, 1997a,b).

In addition to membrane-bound regulators, soluble factor H is an efficient regulator of the activity of the alternative pathway C3 and C5 convertases in plasma and on host cell surfaces (Weiler *et al*, 1976; Fearon and Austen, 1977; Whaley, 1980). Down-regulation of C activation by factor H occurs when it binds to host cells that carry deposited C3b molecules and anionic structures like sialic acids and glycosaminoglycans (Fearon, 1978; Pangburn and Müller-Eberhard, 1978; Meri and Pangburn, 1990). Factor H is translated from an mRNA of 4.4 kb to a 150 kDa protein that is composed of 20 domains called short consensus repeats (SCR) (Kristensen *et al*, 1986). In factor H three C3b binding sites and 2–3 polyanion binding sites are distributed along the approximately 600 Å long molecule (Pangburn *et al*, 1991; Sharma and Pangburn, 1996; Blackmore *et al*, 1998; Jokiranta *et al*, 2000). Factor H inhibits the alternative C pathway by preventing factor B binding to C3b, promoting dissociation of the C3bBb enzyme complex (decay accelerating activity) and by acting as a cofactor for factor I-mediated inactivation of C3b.

Recent studies have utilised a novel mAb-based immunoassay (BTA-TRAK) for the detection of urinary bladder cancer (Heicappel *et al*, 1999; Raitanen *et al*, 2001). The mAb used in the assay recognises factor H or a factor H-related molecule in the urine of patients with bladder cancer (Kinders *et al*, 1998). Factor H is the prototype molecule of a family of factor H-related molecules (Zipfel *et al*, 1999). The family includes factor H, factor H-like protein 1 (FHL-1) and factor H-related proteins (FHRs) 1, 2, 3, 4 and 5. It has been observed recently that malignant glioblastoma cells produce factor H and FHL-1, the only two proteins in this family with a clearly documented C inhibitory function (Gasque *et al*, 1996; Junnikkala *et al*, 2000).

FHL-1 is an alternatively spliced product of the factor H gene, composed of seven N-terminal SCRs of factor H plus an additional four amino acids (Hellwage *et al*, 1997; Zipfel and Skerka, 1999). FHL-1 has essentially the same C inhibiting functions (cofactor activity, inhibition of factor B binding and decay accelerating activity) as factor H (Kühn *et al*, 1995; Kühn and Zipfel, 1996; Hellwage *et al*, 1997). In the present study our aim was to examine whether human tumour cells secrete soluble C inhibitors that promote C3b inactivation. When several cell lines were examined FHL-1 and factor H were found to be secreted by the ovarian tumour cells SK-OV-3 and Caov-3 and by the erythroleukaemia cell line K562. Two ovarian tumour cell lines that did not synthesize factor H or FHL-1 produced a soluble form of MCP/CD46 with a partially similar functional profile as factor H. Immunohistological analyses showed the presence of FHL-1 and factor H on ovarian tumours *in vivo*. Factor H and FHL-1 were also abundant in ascites fluid samples of patients with ovarian cancer. Notably, the level of FHL-1 was disproportionately high when compared to other biological fluids.

These results indicate that ovarian tumour cells produce factor H/FHL-1, or soluble MCP that may suppress C activation within the tumour microenvironment.

## MATERIALS AND METHODS

### Cell lines

The SK-OV-3, Caov-3, PA-1 and SW626 cell lines were originally obtained from American Type Culture Collection (ATCC, Manassas, VA, USA). The H2 glioma cell line was established from a human glioblastoma (Jääskeläinen *et al*, 1989). Neuronal cell lines SY5Y and Paju were obtained from Dr Y Shen (Sun Health Research Institute, Phoenix, AZ, USA) and from Professor LC Andersson (Haartman Institute, Helsinki, Finland), respectively. The HF-1 lymphoma cell line was from Dr M Kaartinen (Haartman Institute, Helsinki, Finland). Other cell lines listed in Table 2

**Table 2:**
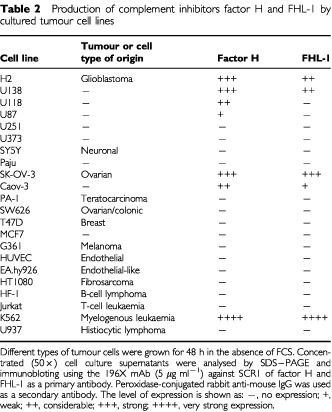
Production of complement inhibitors factor H and FHL-1 by cultured tumour cell lines

were from ATCC. All cell lines were grown in RPMI 1640 medium (Gibco Laboratories, Paisley, UK) supplemented with 10% (v v^−1^) heat-inactivated foetal calf serum (FCS) (Gibco), penicillin (10 U ml), streptomycin (100 μg ml^−1^) and 2 mM L-glutamine (Nord Cell, Bromma, Sweden).

### Ascites and tumour samples

Ascites fluid (AF) samples (*n*=16) were obtained in a prospective, nonselective fashion from previously untreated patients with ovarian cancer at the Department of Obstetrics and Gynecology (Haukeland Hospital, Bergen, Norway). The samples were obtained during laparotomy immediately after the peritoneal cavity was opened. The median age of the patients was 63.5 years (range from 30–75 years) and the clinical stages were determined according to the FIGO criteria. AF samples from patients with liver cirrhosis were collected from patients treated at the Transplantation and Liver Surgery Unit (Helsinki University Hospital, Helsinki, Finland). Two control samples of fluids from ovarian follicles were obtained during laparoscopy and draining of follicular cysts. Ovarian tumour samples (*n*=25) were obtained from patients treated at the Department of Obstetrics and Gynecology, Helsinki University Central Hospital, Helsinki, Finland. The study protocols were approved by the Ethical Committees of the Haukeland Hospital, the Helsinki University Central Hospital and by the Regional Ethical Committee of Western Norway (Jnr. 234/97-81.97).

### Antibodies

Polyclonal goat anti-factor H antibodies were purchased from Incstar Co. (Stillwater, MO, USA) or from Calbiochem (La Jolla, CA, USA). A cell line producing the mAb 196X that binds to the SCR1 domain of factor H-/FHL-1 was originally from J Tamerius (Quidel Corp., San Diego, CA, USA) and grown as described (Jokiranta *et al*, 1996). The mAb VIG8 against the C-terminal SCR19-20 domain of factor H was obtained from Dr W Prodinger (Prodinger *et al*, 1998). The mouse anti-CD46 mAb GB24 (IgG1) was kindly provided by Dr K Liszewski and Professor JP Atkinson (Washington University School of Medicine, St. Louis, MO, USA). An irrelevant mouse IgG (16.1.2 anti-idiotype mAb) was obtained from Dr M Kaartinen (Haartman Institute, Helsinki, Finland).

### RT–PCR analysis

Total RNA was extracted from the cultured ovarian tumour cells using Trizol LS Reagent (Gibco), as recommended by the manufacturer. Isopropyl alcohol precipitated RNA was dissolved in diethylpyrocarbonate-treated water and 1 μg of RNA was denatured at 70°C for 10 min and then immediately chilled on ice. The reverse transcriptase reactions were carried out using RNA in a total volume of 20 μl of reverse transcriptase buffer (Gibco) containing 10 mM dithiotreitol (Gibco), 500 μM dNTP Mix (Pharmacia), 25 μg ml^−1^ oligo(dT)12-18 (Pharmacia) and 200 U Moloney murine leukaemia virus reverse transcriptase (Gibco). The reaction was allowed to occur for 1 h at 37°C.

Each PCR reaction was carried out in a 100 μl volume containing 200 μM dNTP (Pharmacia), 10 pmol of each of the specific primers and 2.5 U Taq polymerase (Pharmacia) in a PCR buffer containing 1.5 μM MgCl_2_. The samples were denatured at 94°C for 5 min and amplification was performed on a Perkin Elmer GeneAmp PCR System 2400, with denaturation at 94°C for 1 min, annealing at 46°C for 1 min and extension at 72°C for 1 min for 30 cycles. The final reaction step was followed by a 10-min extension step at 72°C to ensure that the amplified DNA was double-stranded. To confirm the absence of contaminants, negative controls were included in each RT–PCR assay, in which the RNA samples were replaced by sterile water or the reverse transcriptase was omitted. Amplified products (10 μl for each PCR sample) were electrophoresed in parallel with size markers (Gibco) on a 1% agarose gel. The gels were stained with ethidium bromide and photographed under UV light. The identities of the PCR products were confirmed by cloning into the pCRII TA cloning vector (Invitrogen, Groningen, The Netherlands) and sequencing. The PCR primers used have been described earlier (Junnikkala *et al*, 2000).

### Immunoblotting and ELISA

The cell lines were grown in RPMI without FCS for 48 h to obtain serum-free growth supernatants. The supernatants were concentrated 50-fold with the Millipore Ultrafree®-15 Centrifugal Filter Device (Bedford, MA, USA). Aliquots of concentrated supernatants, ascites samples, control samples, NHS and ovarian follicle fluids (all diluted 1/50 except the supernatants) were electrophoresed on a 10% SDS–PAGE slab gel under nonreducing conditions and transferred to a nitrocellulose filter with a pore size of 0.25 μm (Schleicher & Schuell, Dassel, Germany). After blocking nonspecific binding sites with 5% human milk/PBS the filter was incubated first with an anti-factor H mAb (196X; 5 μg ml^−1^) overnight at 4°C. After washing peroxidase-conjugated rabbit anti-mouse IgG (diluted 1 : 2000) (Jackson ImmunoResearch, West Grove, PA, USA) was added and incubated for 1 h at 22°C. For detection of MCP/CD46 the GB24 mAb was used under similar conditions. The filters were washed twice and the bound antibodies were visualised using an ECL Western blotting kit (Amersham Life Sciences, Amersham, UK). To estimate the ratios between factor H and FHL-1 in the samples the immunoblots were analysed by density scanning using the MacBAS v2.5 programme (Fuji Photo Film Co., Ltd, Japan).

To determine the amounts of factor H and FHL-1 in the ascites samples an ELISA assay was set up. Microtiter (Nunc Polysorp, Denmark) plates were coated with a polyclonal goat-anti-human fact or H antibody (Calbiochem, La Jolla, CA, USA) diluted in carbonate buffer (15 mM Na_2_CO_3_, 35 mM NaHCO_3_, pH 9.6). After an overnight incubation at +4°C the wells were washed with PBS/Tween 0.05% and nonspecific binding sites were blocked by incubation with 5% BSA/PBS for 1 h at +37°C. The plates were washed and the samples were applied in appropriate dilutions in 0.5% BSA/PBS. For a standard curve purified factor H was added in four dilutions. After a 2 h incubation the plates were washed and the monoclonal anti-factor H antibody 196X, 3 μg ml^−1^ in 0.5% BSA/PBS, was added and incubated for 3 h at +37°C. 196X binds to SCR1 of both factor H and FHL-1. After washing the secondary antibody, HRP-conjugated rabbit-anti-mouse IgG (Jackson), diluted 1 : 2000 in 0.5% BSA/PBS plus 1% goat serum was added to the wells. After incubation (2 h, 37°C) the plates were washed and the substrate was added. The colour reaction was stopped with 2M H_2_SO_4_ and the absorbances were measured at 492 nm with a Wallac Victor Multilabel Counter (Wallac, Turku, Finland).

### Immunoperoxidase staining of ovarian tumour samples

Serial cryostat sections (4–5 μm) were prepared from fresh ovarian tumour tissue samples that had been frozen in isopentane, cooled with liquid nitrogen and stored at −70°C. The sections were allowed to attach to poly-L-lysine coated glass slides, air dried and fixed in cold (−20°C) acetone for 5–10 min. The primary mAbs (VIG8, 196X, GB24) were applied on the sections at 5 μg ml^−1^ and incubated overnight at room temperature. An irrelevant mouse IgG (16.1.2 anti-idiotype mAb) was used as a control. The sections were washed, incubated for 30 min with HRP-conjugated anti-mouse immunoglobulins and stained according to the manufacturer's instructions (Vectastain Elite® ABC Kit, Vector Laboratories, Inc., Burlingame, CA, USA). After washing, the sections were treated with the enzyme substrate aminoethylcarbazol and counterstained with Mayer's haematoxylin.

### Binding of radiolabelled FHL-1 and factor H to ovarian tumour cells

Complement factor H (obtained from Calbiochem or purified as described) (Meri and Pangburn, 1990) and recombinant FHL-1 (rFHL-1; produced and purified as earlier described) (Kühn *et al*, 1995) were radiolabelled with Na^125^I (NEN™, Boston, MA, USA) using an Iodogen method according to the manufacturer's instructions. Free ^125^I was separated from the labelled proteins by gel filtration through Sephadex G25 (Pharmacia). Specific activities of the radiolabelled factor H and rFHL-1 proteins were 6.6×10^7^ c.p.m. μg^−1^ and 3.3×10^6^ c.p.m. μg^−1^, respectively. Binding of rFHL-1/factor H to ovarian cells was examined by incubating triplicate samples of 3×10^5^ of cells with fixed or varying amounts of ^125^I-labelled factor H or FHL-1 as indicated in the respective figure legends. In inhibition experiments, 3–100-fold higher amounts of unlabelled factor H or BSA were incubated with cells together with ^125^I-labelled factor H (30 ng). Briefly, the mixtures were incubated for 1 h at 37°C with continuous shaking in a final volume of 120 μl of GVBS (0.1% gelatin, veronal buffered saline, pH 7.4) diluted 1 : 3, layered on top of a 250-μl cushion of 20% sucrose in narrow 0.4-ml test tubes and centrifuged for 5 min at 6100 **g**. Cell pellets were cut off from the tubes. Both pellets and supernatants were counted for radioactivity and the binding percentages were determined as the proportion of cell-bound *vs* total radioactivity. All binding experiments were performed twice.

### Assay for cofactor activity in ovarian cell growth supernatants

Complement C3b was purified as described (Koistinen *et al*, 1989) and radiolabelled with ^125^I to a specific activity of 1.7×10^7^ c.p.m. μg^−1^. Nonincorporated ^125^I was separated from the labelled protein by gel filtration through Sephadex G25 (Pharmacia). Cofactor activity of cell growth supernatants for C3b cleavage was examined as described earlier (Junnikkala *et al*, 2000). Briefly, 10 μl portions of 50×-concentrated supernatants were incubated (2 h, 37°C) with 10 ng (10^5^ c.p.m.) of ^125^I-labelled C3b and 3 μg of factor I (Calbiochem) in a total volume of 30 μl and subjected to SDS–PAGE and autoradiography. As a control the inactivations were carried out in the absence of factor I and/or cell growth supernatant and no ^125^I-C3b cleavage was observed. In subsequent experiments the supernatants were first preincubated with a polyclonal anti-factor H antibody (50 μg ml^−1^) and/or with the GB24 anti-MCP (25 μg ml^−1^) mAb (30 min, 37°C) or buffer (control) before incubation with ^125^I-C3b and factor I (90 min, 37°C). As a positive control ^125^I-C3b and factor I were incubated with factor H (25 μg ml^−1^).

## RESULTS

### FHL-1 and factor H in ascites fluids of ovarian cancer patients

**Figure 1:**
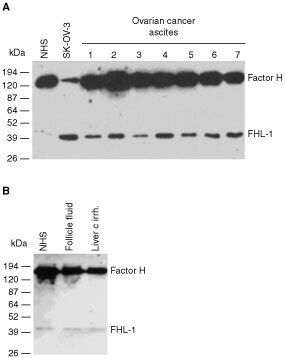
Detection of FHL-1 and factor H in ascites fluids of ovarian cancer patients. (**A**) Ascites fluid samples (diluted 1 : 50) were obtained from patients with ovarian tumours and NHS from a healthy control subject. The samples were analysed by SDS–PAGE and immunoblotting using the 196X mAb that recognises SCR1 of factor H and FHL-1 as the primary antibody. Histological types of the ovarian tumour samples of the patients were as follows: 1, serous cystadenocarcinoma; 2, papillary serous cystadenocarcinoma; 3, endometrioid adenocarcinoma; 4, undifferentiated adenocarcinoma; 5, mucinous adenocarcinoma; 6, serous cystadenoma; 7, adenocarcinoma from the left ovarian tube. In (**B**) NHS, an ovarian follicle fluid sample and ascites from a patient with liver cirrhosis (all diluted 1 : 50) were similarly analysed. In all ovarian tumour samples the relative proportional of FHL-1 was higher than in control samples.

The current study was initiated by analysis of soluble C inhibitors factor H and FHL-1, the short truncated form of factor H, in ascites fluid (AF) samples of patients with ovarian cancer. Immunoblotting with the 196X mAb (recognizing SCR1 of both factor H and FHL-1) (Figure 1

) suggested that most of the ascites samples (*n*=16) examined contained higher levels of factor H and especially of FHL-1 than NHS. Control fluid samples from two ovarian follicle cysts were found to contain similar amounts of factor H and FHL-1 as NHS (Figure 1B).

In an ELISA assay, employing the 196X mAb, the combined mean level of factor H and FHL-1 was higher in the cancer patient AF (758±312 μg ml^−1^, mean±s.d.; *n*=16) than in NHS (461±63 μg ml^−1^, *n*=8; Student's *t*-test, *P*<0.05) or in AF samples from liver cirrhosis patients (43±27 μg ml^−1^, *n*=6). These results showed that the potent C inhibitors factor H and FHL-1 were abundantly present in the AF of patients with ovarian cancer and that the relative proportion of FHL-1 was clearly increased in the malignant ascites specimens. Using the ELISA assay and quantitative density scanning, the mean amounts of FHL-1 were found to be considerably higher in the AF samples from ovarian cancer patients (130 μg ml^−1^; FHL-1/factor H ratio 17.5%) than in NHS (24 μg ml^−1^, 5.2%; *P*<0.01), in the AF samples from patients with liver cirrhosis (<2 μg ml^−1^, <5.0%) or in the follicle fluids (18 μg ml^−1^, 5.0%), respectively.

### Immunohistochemical detection of FHL-1 and factor H in ovarian tumours

**Figure 2:**
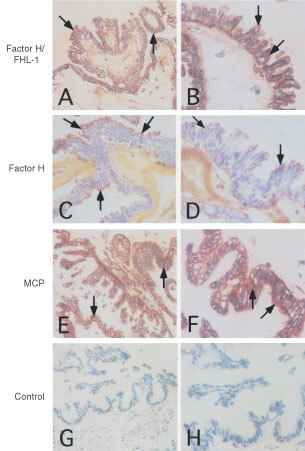
Immunohistochemical analysis of the presence of FHL-1 and factor H in ovarian tumours. Cryostat sections (5 μm) of a serous cystadenocarcinoma were fixed and stained with the 196X (**A**, **B**) and VIG8 (**C**, **D**) mAb against factor H/FHL-1 SCR1 and factor H SCR19-20, respectively. MCP expression was analysed by the mouse anti-MCP mAb BG24 (**E**, **F**) and an irrelevant mouse IgG was used as a negative control (**G**, **H**). The bound mAbs were detected using the Vectastain ABC immunoperoxidase staining kit. Original magnifications, 200× (left row) and 400× (right row). While combined staining for factor H and FHL-1 occurred throughout the tumour epithelium (**A**, **B**) positive staining for factor H (**C**, **D**) was seen most strongly on the outermost mucus layer. Arrows indicate positively stained areas.

To investigate the presence of FHL-1 and factor H in ovarian tumours *in vivo*, we analysed cryostat sections of serous ovarian tumours with the VIG8 (detects SCR19-20 of factor H) and 196X (detects SCR1 of factor H and FHL-1) mAbs and immunoperoxidase staining. Examples of the stainings are shown in Figure 2

. The possible presence of FHL-1 was judged by a comparison of staining with the 196X mAb (Figure 2Aand B) to staining with the VIG8 mAb. The staining with 196X showed a similar pattern as VIG8, but a stronger signal was seen through all layers of the epithelial tumour cells. Factor H (VIG8 positive staining) was detected mostly in the apical layers of the tumour cells but to a lesser extent also on the extracellular matrix (Figure 2Cand D). Staining with the GB24 anti-MCP mAb showed a strong positive staining throughout the tumour cells (Figure 2Eand F). An irrelevant mouse IgG (negative control) showed only a week nonspecific staining of fibroblasts, collagen fibrils and blood vessels in the submucosa (Figure 2G and H). An analysis of a series of serous ovarian carcinoma samples (*n*=25) demonstrated that all tumours expressed factor H/FHL-1. The level of expression, however, ranged from weak to strong. The expression was weak, considerable or strong in 11, 12 or 2 samples, respectively. In addition to cellular staining, factor H/FHL-1 was detected also on the extracellular matrix. MCP was expressed by all the tumours, expression being strong in 18 out of the 25 tumours. Factor H/FHL-1 expression was strongest in tumours with the largest size but otherwise no apparent correlations to clinical parameters were observed (Table 1

**Table 1:**
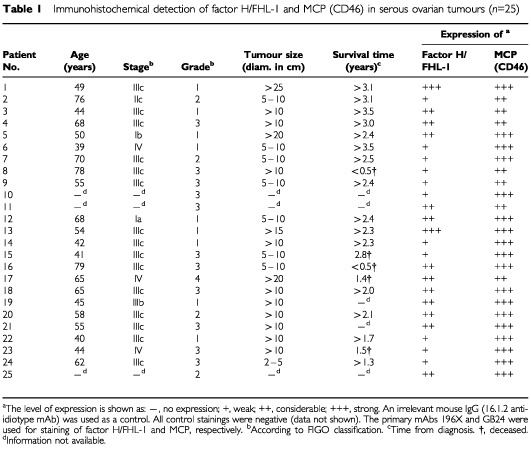
Immunohistochemical detection of factor H/FHL-1 and MCP (CD46) in serous ovarian tumours (*n*=25)

). Together with the data obtained from the immunoblotting and ELISA analyses of ascites samples (Figure 1) the immunohistochemical observations suggested that ovarian tumour cells are capable of producing FHL-1 and factor H into their microenvironment.

### Production of factor H and FHL-1 by ovarian tumour cell lines

**Figure 3:**
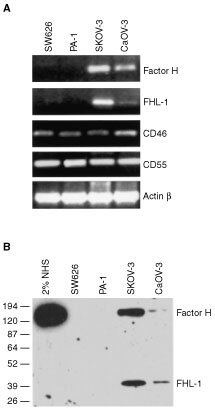
Expression of C regulators factor H and FHL-1 by ovarian tumour cell lines. (**A**) RT–PCR analysis of mRNA expression of FHL-1, factor H, MCP (CD46) and DAF (CD55) by the cell lines SW626, PA-1, SK-OV-3 and Caov-3. mRNAs were isolated from the tumour cell lines and analysed by RT–PCR, β-actin mRNA expression was tested as a positive control. Factor H and FHL-1 mRNAs were detected in SK-OV-3 and Caov-3 cells but not in the two other cell lines. (**B**) Analysis of FHL-1 and factor H protein production by SW626, PA-1, SK-OV-3 and Caov-3 cells. Growth supernatants of cells grown for 48 h in the absence of FCS were collected and concentrated 50-fold. NHS (2%) was used as a positive control. The samples were run in SDS–PAGE under nonreducing conditions and analysed by immunoblotting. 196X mAb (5 μg ml^−1^), which detects both factor H and FHL-1, was used as the primary antibody, and peroxidase-conjugated rabbit anti-mouse IgG as the secondary antibody. Molecular weights of the marker proteins are indicated on the left. Note higher FHL-1/factor H ratios in the ovarian tumour cell supernatants as compared to NHS.

To analyse the production of factor H/FHL-1 by cultured tumour cells we first examined a panel of several cell lines. When a total of 22 cell lines were tested only the ovarian cell lines SK-OV-3 and Caov-3, the glioma cell lines H2 and U138 and the K562 erythroleukaemia/lymphoblast cells constitutively produced factor H and FHL-1 to the culture medium (Table 2). Two cell lines (U118 and U87) produced only factor H. This result showed that the production of factor H and FHL-1 is not a ubiquitous property of all tumour cells, but restricted to certain tumour cell types. When the ovarian tumour cells were analysed by RT–PCR the SK-OV-3 and Caov-3 cell lines were found to express factor H and FHL-1 mRNAs, although the amount of FHL-1 mRNA was relatively low in the Caov-3 cells. The sizes of factor H (4.4 kb) and FHL-1 (1.8 kb) mRNAs in these cells were identical to those detected in human liver tissue. In contrast, the PA-1 and SW626 cell lines were negative for both factor H and FHL-1 mRNA expression (Figure 3A

). All cell lines expressed mRNAs for the two membrane regulators of complement CD46 (MCP) and CD55 (DAF) which both regulate C activation at the C3 level (Figure 3A).

In accordance with the RT–PCR analyses, immunoblotting with the 196X mAb showed that SK-OV-3 and Caov-3 cells constitutively produced and secreted both the 150 kDa factor H and the 42 kDa FHL-1 protein to an FCS-free culture medium during a 48 h incubation. The other two cell lines PA-1 and SW626 cells remained negative for factor H/FHL-1 production (Figure 3B). At the protein level both SK-OV-3 and Caov-3 cells produced approximately similar amounts of FHL-1 and factor H. This is in contrast with the situation in serum where the concentration of factor H is over 10 times higher than that of FHL-1 (Zipfel *et al*, 1999).

### Binding of factor H and FHL-1 to ovarian tumour cells

**Figure 4:**
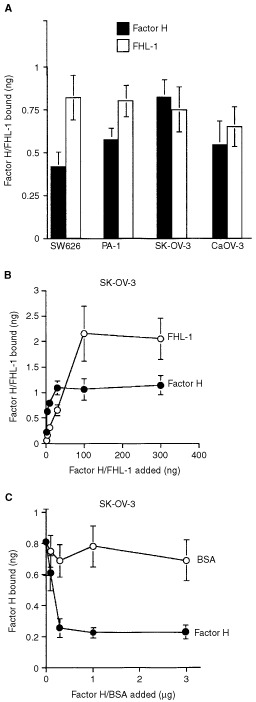
Factor H/FHL-1 binding to ovarian tumour cells. (**A**) Binding of ^125^I-labelled factor H and FHL-1 to ovarian tumour cells SW626, PA-1, SK-OV-3 and Caov-3. Cells (93×10^5^) were incubated with radiolabelled factor H (30 ng, 6.6×10^6^ c.p.m. μg^−1^) or FHL-1 (30 ng, 3.3×10^6^ c.p.m. μg^−1^) for 1 h at 37°C in 1 : 3 GVBS, pH 7.4. The results (mean±s.d.; *n*=3) are expressed as the amount of bound protein (ng). A representative of three experiments with similar results is shown. (**B**) Dose-response analysis of binding of ^125^I-labelled factor H and FHL-1 to SK-OV-3 cells. (**C**) Inhibition analysis of binding of ^125^I-labelled factor H to SK-OV-3 cells. Indicated amounts of unlabelled factor H or BSA (up to a 100-fold excess) were incubated together with ^125^I-labelled factor H as in (**A**).

The immunohistochemical evidence of tumour-associated factor H/FHL-1 suggested that the ovarian tumour cells could carry surface components that bind factor H and/or FHL-1, proteins which both have binding sites for glycosaminoglycans and other polyanions. In accordance, we found that all the ovarian tumour cell lines SK-OV-3, Caov-3, PA-1 and SW626 bound both ^125^I-labelled factor H and FHL-1 to their cell surfaces (Figure 4A

). Binding of factor H was most efficient to the SK-OV-3 cells and occurred in a saturable manner (Figure 4B). The binding of ^125^I-factor H could be inhibited maximally by 70% with unlabelled factor H (Figure 4C). The binding data indicated that the ovarian cells SK-OV-3, Caov-3, PA-1 and SW626 have the ability to bind soluble factor H and FHL-1 from the fluid phase even in the absence of initial C3b deposition.

### Cofactor activity for C3b inactivation in ovarian tumour cell supernatants

**Figure 5:**
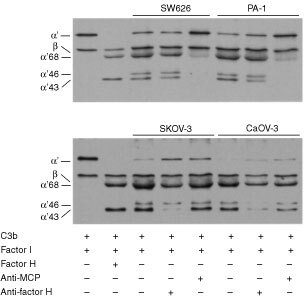
Cofactor activity of the ovarian cell growth supernatants for factor I-mediated cleavage of ^125^I-labelled C3b. Cell supernatants (concentrated 50-fold) were first preincubated with or without polyclonal anti-factor H (50 μg ml^−1^) or the GB24 anti-MCP (25 μg ml^−1^) antibody (37°C, 30 min) and then for 90 min at 37°C with ^125^I-labelled C3b and factor I (25 μg ml^−1^). In the positive controls ^125^I-C3b and factor I were incubated with factor H (25 μg ml^−1^). The mixtures were analysed by SDS–PAGE and autoradiography under reducing conditions. The protein compositions in the mixtures are marked beneath the lanes. The α′-chain of ^125^I-C3b becomes cleaved into fragments with apparent M_r_s of 68, 46 and 43 kDa. Because of the relatively high concentration of factor H in the positive control (second lane from the left) the second factor I-mediated cleavage had fully converted the 46 kDa fragment into the 43 kDa α′-chain fragment. The cofactor activity of the SW626 and PA-1 supernatants could be inhibited with the anti-MCP mAb whereas the activities of the SK-OV-3 and Caov-3 supernatants were inhibited with the polyclonal anti-factor H antibody. The results indicate that all the ovarian cell supernatants have cofactor activity for C3b cleavage. In SK-OV-3 and Caov-3 cells this is due to functionally active H/FHL-1, whereas in the SW626 and PA-1 cell supernatants it is due to soluble MCP.

Since SK-OV-3 and Caov-3 cells were found to produce and secrete both factor H and FHL-1 into their growth supernatants we wanted to study whether they were capable of promoting the degradation of C3b to iC3b, i.e. whether the secreted proteins acted as cofactors for factor I. Cofactor activity analysis demonstrated that the supernatants of all the cell lines, including those of PA-1 and SW626, promoted factor I-mediated cleavage of ^125^I-labelled C3b (Figure 5

). When the supernatants were preincubated with a polyclonal anti-factor H antibody the cofactor activities of SK-OV-3 and Caov-3, but not of PA-1 and SW626 cell supernatants were clearly reduced. The fact that PA-1 and SW626 cells did not produce factor H or FHL-1, yet their growth supernatants promoted C3b degradation suggested that other cofactors for factor I could be involved. The explanation to this was suggested by immunoblotting analysis which showed that PA-1 and SW626 cells produced a soluble form of the membrane cofactor protein (MCP/CD46) into their growth supernatants (data not shown). The role of MCP was verified by studies with the anti-MCP mAb GB24, which completely blocked the cofactor activities of PA-1 and SW626 cell supernatants (Figure 5). No CR1, another potential cofactor candidate for C3b inactivation, was detected in any of the cell supernatants (data not shown). In summary, the results indicated that all the four cell lines secreted cofactor activity for factor I. In two cases (SK-OV-3 and Caov-3) this was mainly due to factor H and FHL-1 and in the other two cases (PA-1 and SW626) it was due to a soluble form of MCP.

## DISCUSSION

In this study, we observed that ovarian tumour cells can secrete and/or utilise functionally active inhibitors of the cytolytic C system, factor H and FHL-1. This suggests that ovarian tumours create a surrounding microenvironment, where fluid phase C activation may be inhibited. The abundant presence of factor H and FHL-1 in the AF samples and in the apical layers and extracellular matrix surrounding the tumour cells of patients with ovarian carcinoma suggest that C activation is controlled within the peritoneal cavity. This is one reason to explain why neither the antibody-initiated classical pathway nor the alternative pathway is effective in destroying ovarian tumour cells.

We have recently demonstrated that the C resistant H2 glioblastoma cell line constitutively produced factor H and FHL-1 and protected itself against C attack *in vitro* (Junnikkala *et al*, 2000). A survey of several cell lines showed that the production of these C regulators is not restricted to glioblastomas but occurs also in ovarian tumour cells and in the K562 erythroleukaemia cells (Table 2).

There is already some evidence for the role of factor H in the resistance of nucleated tumour cells against C damage initiated by the classical pathway (Ollert *et al*, 1995). In accordance with our studies, certain glioma and neuroblastoma cells have been shown to express factor H mRNA (Gasque *et al*, 1992, 1996) and a human oligodendrocyte cell line (HOG) secreted factor H when stimulated with interferon-γ (Gasque and Morgan, 1996). There is also evidence that factor H can bind to polymorphonuclear leucocytes (PMN), U937 cells and B lymphoblastoid Raji cells (Erdei and Sim, 1987; Avery and Gordon, 1993; Discipio *et al*, 1998). However, the current study is the first one to propose such a role for FHL-1 and to show the presence of FHL-1 in samples from patients with ovarian tumours. Since FHL-1 shares the C regulatory functions with factor H (Friese *et al*, 1999b) it can be proposed that it perhaps controls C activation within the microenvironment of tumour cells.

The amounts of FHL-1 in ascites samples from ovarian cancer patients were found to be considerably higher than in NHS or in ascites from a patient with liver cirrhosis (Figure 1). The fact that they were also higher in AF than in ovarian follicle fluids suggests that production of FHL-1 is related to malignancy rather than to the source of cells. As the patients (*n*=16) from whom the samples were obtained had different types of ovarian tumours and samples from all of them were positive for FHL-1 it is likely that the production of FHL-1 is a general feature of ovarian tumours. However, there were up to 3–4-fold differences in the amounts of factor H/FHL-1 between the AF samples of ovarian cancer patients. Whether these are individual differences between the patients or whether a high expression level is related to certain ovarian tumour types remain to be further studied. Since ascites represents a separate fluid compartment within the peritoneal cavity, and the tumour cells are in direct contact with the AF, it is probable that FHL-1 and factor H synthesised by the tumour cells are concentrated into the peritoneal cavity and may protect tumour cells locally against C-mediated damage.

To further investigate the expression of FHL-1 and factor H *in vivo* we performed immunohistological analysis of tumour tissue samples obtained from 25 patients with serous cystadenocarcinoma, the most common type of malignant ovarian neoplasm (Christopher, 1994). Table 1 summarizes the expression levels of factor H/FHL-1 and MCP and demonstrates that the expression of MCP was strong in most of the tumours. This indicates that MCP is a common, strongly expressed regulator in ovarian tumours. The staining intensity of factor H/FHL-1 varied from weak to strong being considerable in most of the cases. Staining with the 196X mAb that detects both factor H and FHL-1 showed a stronger positive signal (Figure 2A and B) than staining with the VIG8 mAb, which detects only factor H (Figure 2C and D). Staining for factor H/FHL-1 was seen in both the apical tumour cell layers and in the intercellular spaces. It is thus likely that both factor H and FHL-1 bind to the apical epithelium. The proteins can be directly produced by the tumour cells and/or they can infiltrate from the blood to the ascites and then bind to the apical surfaces of tumour cells. Since both proteins were found in the apical tumour cell layers (Figure 2A–D), it can be suggested that these layers form a protective barrier against C attack. The data on immunoblotting (Figure 1) and ELISA analysis of ascites samples further supported the immunohistological results and indicated that the ovarian tumour cells are capable of producing FHL-1 and factor H *in vivo*.

RT–PCR (Figure 3A) and immunoblotting (Figure 3B) experiments showed that the SK-OV-3 and Caov-3, but not the PA-1 and SW626, ovarian tumour cell lines produced factor H and FHL-1 mRNA and protein. The expression of factor H/FHL-1 was constitutive and did not require exogenous stimuli. There is evidence that this is the case also with human fibroblasts and HUH7 hepatic (Friese *et al*, 1999a), HTB153 rhabdomyosarcoma (Legoedec *et al*, 1995) and CB193 astroglioma cells (Gasque *et al*, 1995). These results suggest that, although not ubiquitous, the production of factor H and FHL-1 may be a relatively common C resistance mechanism of tumours of different origin than previously thought.

The relatively high proportion of FHL-1, as compared to factor H, in the SK-OV-3 and Caov-3 cell growth supernatants (Figure 3B) suggested that FHL-1 may have a role in the control of the alternative C pathway activation against tumour cells. The average FHL-1/factor H ratio in the ovarian cancer patient AF samples was found to be over three-fold higher than in NHS or in follicle fluids (17.5% *vs* 5.2% or 5.0%, respectively). FHL-1 thus appears to be preferentially produced by malignant cells also *in vivo*.

SK-OV-3, Caov-3, PA-1 and SW626 ovarian tumour cells were found to bind both ^125^I-labelled factor H and FHL-1 to their cell surfaces (Figure 4). This suggested that the surfaces of cultured ovarian cells have structures that bind factor H and FHL-1 from the surrounding medium or from plasma. The relatively high number of factor H and FHL-1 molecules bound to the tumour cells, approximately 10^4^ and 5×10^4^ per cell, respectively, is probably due to an abundancy of low affinity receptors, e.g. glycosaminoglycans or sialic acid-type polyanions on the cell surfaces.

To verify that factor H and FHL-1, that are produced by the SK-OV-3 and Caov-3 cells and bind to them, were functionally active we tested whether the growth supernatants of these cells could promote factor I-mediated cleavage of ^125^I-labelled C3b to its inactive form iC3b. Both SK-OV-3 and Caov-3 cell supernatants promoted factor I-mediated cleavage of C3b to iC3b (Figure 5). This activity was inhibited by a polyclonal antibody against factor H. Surprisingly, also the supernatants of PA-1 and SW626 cells promoted C3b cleavage. The reason for this was revealed when we found out that these cell lines produced soluble MCP (Hakulinen *et al*, unpublished results) and that it was possible to inactivate the cofactor activity with the GB24 anti-MCP mAb (Figure 5). Earlier, soluble forms of MCP have been detected in body fluids (Hara *et al*, 1992) and also in cancer patients' sera that contained increased amounts of the 56 and 47 kDa soluble forms of MCP (Seya *et al*, 1995).

The different behaviour of PA-1 and SW626 cells may be related to their possibly different origins as compared to the SK-OV-3 and Caov-3 cells. PA-1 is a teratocarcinoma cell line and for SW626, although it has been isolated from an ovarian tumour, recent observations suggest that it could originate from a colon tumour metastasis (Furlong *et al*, 1999).

Taken together, our observations indicate that the ovarian tumour cell lines have similar strategies, but different factors, to suppress C activation on their surfaces. In addition to the cell membrane regulators, it is possible that factor H, FHL-1 and soluble MCP may restrict C activation and prevent deposition of the terminal C components to the surfaces of tumour cells. Production of C inhibitors by tumour cells can be an important factor also when tumour treatment with C activating mAbs is considered (Gorter and Meri, 1999; Harjunpää *et al*, 2000). In the case of ovarian carcinoma, the presence of the tumour cells within the peritoneal cavity makes them an attractive target for local immunotherapy. Such a therapy could be of considerable benefit if the activity of the C system within the peritoneal space and around the tumours could be enhanced, e.g. by temporarily blocking C inhibitors.
